# Antimicrobial Photodynamic Therapy against *Escherichia coli* and *Staphylococcus aureus* Using Nanoemulsion-Encapsulated Zinc Phthalocyanine

**DOI:** 10.3390/microorganisms11051143

**Published:** 2023-04-27

**Authors:** Nada T. Felifel, Mahmoud A. Sliem, Zienat Kamel, Joanna Bojarska, Mohamed G. Seadawy, Rehab M. Amin, Sherif M. Elnagdy

**Affiliations:** 1 Botany and Microbiology Department, Faculty of Science, Cairo University, Gamma St., Giza 12613, Egypt; 2 National Institute of Laser Enhanced Sciences (NILES), Cairo University, Giza 12613, Egypt; 3 Faculty of Chemistry, Institute of General and Ecological Chemistry, Lodz University of Technology, Żeromskiego 116, 90-924 Lodz, Poland; 4 Biological Prevention Department, Ministry of Defense, Cairo 11766, Egypt

**Keywords:** antimicrobial photodynamic therapy, *E. coli*, multidrug-resistant microorganisms, *S. aureus*, Zn phthalocyanine

## Abstract

Multidrug-resistant microorganisms have become a significant public health threat, and traditional antibiotics are becoming ineffective. Photodynamic therapy (PDT) is a promising alternative that utilizes photosensitizers and light to produce Reactive Oxygen Species (ROS) that can kill microorganisms. Zinc phthalocyanine (ZnPc) is a promising photosensitizer due to its strong affinity for encapsulation in nanoemulsions and its antimicrobial properties. In this study, nanoemulsion was prepared using Miglyol 812N, a surfactant, and distilled water to dissolve hydrophobic drugs such as ZnPc. The nanoemulsion was characterized by its particle size, polydispersity index, Transmission Electron Microscope and Zeta potential, and the results showed that it was an efficient nanocarrier system that facilitated the solubilization of hydrophobic drugs in water. The use of ZnPc encapsulated in the nanoemulsion produced through the spontaneous emulsification method resulted in a significant reduction in cell survival percentages of gram-positive *Staphylococcus aureus* and gram-negative *Escherichia coli* by 85% and 75%, respectively. This may be attributed to the more complex cell membrane structure of *E. coli* compared to *S. aureus*. This demonstrates the potential of nanoemulsion-based PDT as an effective alternative to traditional antibiotics for treating multidrug-resistant microorganisms.

## 1. Introduction

Common bacterial infections, including cellulitis, abscesses and postsurgical infections, are typically caused by pathogens such as *Staphylococcus aureus* and *Escherichia coli,* which can lead to severe local and systemic complications. *S. aureus* is responsible for a range of illnesses from minor skin infections to life-threatening conditions such as meningitis, pneumonia, endocarditis and sepsis [1]. *E. coli* causes both intestinal and extraintestinal infections, including bloodstream infections, pneumonia, skin abscesses and meningitis [2].

However, over the years, many bacteria have developed resistance to antibiotics, which poses a significant threat to public health [3,4]. As a result, there is an urgent need to discover new methods for eradicating these microorganisms [5].

Photodynamic therapy was identified as a promising technique against bacteria, fungi and viruses, as well as an anticancer therapy, as early as 1990 [6]. Antimicrobial photodynamic therapy (aPDT) has been proposed as a viable, topical and non-invasive alternative to antiseptics, antibiotics and essential oils for controlling infectious microorganisms [7,8].

aPDT uses a non-toxic dye called a photosensitizer (PS) that is activated by light in the presence of molecular oxygen (O2) of foreign cells to produce Reactive Oxygen Species (ROS), which can induce cytotoxic effects [9]. PSs tend to accumulate more rapidly in pathogenic agents than in normal healthy cells, though some may also accumulate in healthy cells. However, the photodynamic effect can precisely target foreign cells by limiting the delivery of the light source [10,11]. PS molecules can undergo two types of reactions, known as Type 1 and Type 2 [12]. In the Type 1 reaction, the PS in its excited triplet state reacts with biomolecules (e.g., proteins, lipids and nucleic acids) to produce free radicals that react with molecular oxygen to generate other ROS. The Type 2 reaction occurs when the PS in its excited triplet state reacts with molecular oxygen to produce highly cytotoxic singlet oxygen, facilitated by the Triplet-triplet annihilation phenomenon. Both reactions occur simultaneously. The multi-target nature of aPDT has been shown to play an important role in reducing the development of resistance [13]. The primary targets of photodynamic action are external microbial structures such as the cell membrane, cell wall and virus capsid and envelope. Since the PS adheres to these structures, it does not need to enter the microorganism to be effective. The ROS formed during the photo-inactivation process also act on molecular targets such as proteins, lipids and nucleic acids, though nucleic acids are only affected when microorganisms are already inactivated [14]. This approach prevents the development of resistance in target microorganisms and provides an advantage over conventional antimicrobials [15,16].

The effectiveness of photoinactivation on gram-positive bacteria such as *S. aureus* and gram-negative bacteria such as *E. coli* is highly influenced by the structure of the photosensitizer (PS). The difference in inactivation between the two is associated with the cell wall structure. Gram-positive bacteria have single cell walls composed of peptidoglycan with teichuronic acids and lipoteichoic, which induce permeability. Conversely, the cell wall of gram-negative bacteria is more complex, comprising the peptidoglycan layer, lipopolysaccharides (which are strongly negatively charged), phospholipids, lipoproteins and proteins [12,17].

Several strategies have been developed to increase the penetration of light-sensitive agents into bacterial cells and improve the effectiveness of aPDT. The first step is the selection of appropriate photosensitizers such as phthalocyanines, which are used as dyes and pigments. They belong to the second PS generation and have an intense blue-green color with strong absorption in the red visible region (approximately 600–760 nm), enabling deeper light penetration, efficient ROS generation, high photostability, low toxicity in the dark and easy chemical modification compared to other photosensitizers [18,19]. The second step to improve aPDT is the preparation of nanoemulsions to encapsulate hydrophobic drugs such as Zn phthalocyanines (ZnPcs) to overcome solubility issues in aqueous mediums [20]. Nanoemulsions consist of two immiscible liquids, such as water and oil, stabilized with an interfacial film consisting of a suitable surfactant and cosurfactant, to form a single phase. Nanoemulsions have been classified into three categories: oil-in-water (O/W) type (where oil is dispersed in the aqueous phase), water-in-oil (W/O) type (where water is dispersed in the oil phase) and multiple emulsion systems [21]. Nanoemulsions have several advantages, including the solubilization of hydrophobic compounds, excellent stability, continuous release, reduced toxicity, high physical attraction to bacterial cells and promotion of drug activity [22].

Therefore, the aim of this study is to assess the effect of antimicrobial photodynamic therapy using Zn phthalocyanine encapsulated in nanoemulsion on gram-positive *S. aureus* and gram-negative *E. coli*.

## 2. Materials and Methods

### 2.1. Materials

#### 2.1.1. Chemical Materials

- Photosensitizer: Zn phthalocyanine was purchased from Sigma Aldrich Co., Ltd. (St. Louis, MI, USA);
- Solvents:
- High-performance liquid chromatography (HPLC) Acetone was purchased from (Loba Chemie laboratory reagents and fine chemicals, Murud, India);
- Tetrahydrofuran (THF) was purchased from (Al-Gomhoria Company for Trade in Medicines, Chemicals, and Medical Supplies, Cairo, Egypt);
- Essential Oil: Miglyol 812N was gifted from Cremer oleo division, Germany;
- Biopolymer surfactants: Poloxamer 188 was purchased from (Sigma Aldrich Co.);
- Lipophilic surfactant: Span 80 was purchased from (Sigma Aldrich Co.);
- Hydrophilic surfactant: Tween 80 was purchased from (Al-Gomhoria Company for Trade in Medicines, Chemicals and Medical Supplies, Cairo, Egypt).

#### 2.1.2. Bacteria Strain and Culture Preparation

The bacterial strains used in this study (*S. aureus* ATTCC6538 and *E. coli* ATTCC8739) were obtained from the Department of Botany and Microbiology, Faculty of Science, Cairo University.

#### 2.1.3. Equipment

- The MICROTRAC MRB instrument (Prague, Czechia) at the NAWAH scientific center, Egypt, was used to measure the particle size distribution (mean size), polydispersity index (PdI), zeta potential and viscosity;
- The Transmission Electron Microscope (TEM) (JEM-HR-2100, Tokyo, Japan) at the National Research Centre (NRC), Cairo, Egypt, was used to characterize the structure and morphology of the nanoemulsion (NE) and Zn Phthalocyanine encapsulated in NE (NE + ZnPc). The total magnification was 8.00 kx, and the accelerating voltage was 200 kV;
- A light system composed of a red diode light (Programmable LED, 600–700 nm) at the National Institute of Laser Enhanced Science (NILES), Cairo University, Egypt, was used to irradiate the photosensitizer;
- The water-miscible solvents (acetone and Tetra Hydro Furan) were evaporated in a rotary evaporator device at the National Research Centre, Cairo, Egypt, after complete homogenization of the nanoemulsion and the nanoemulsion with phthalocyanine;
- The Vortex Heidolph reax top (Schwabach, Germany) at the National Research Centre, Cairo, Egypt, was used to mix every mixture thoroughly.

### 2.2. Methods

#### 2.2.1. Preparation of Nanoemulsion

The nanoemulsion was prepared using a modified combination of two methods [23,24] of spontaneous emulsification. First, an organic solution (1) was prepared by dissolving a mixture of surfactants (0.086 g of lipophilic surfactant span 80 and 0.086 g of hydrophilic surfactant tween 80) in 0.4 g of Miglyol oil and 40 mL of water-miscible solvent (34 mL of HPLC acetone + 6 mL of Tetra Hydro Furan) at 40 °C with magnetic stirring. Next, a homogeneous aqueous solution (2) was formed by dissolving 80 mL of distilled water with the biopolymer poloxamer 188 with magnetic stirring at 40 °C. The nanoemulsion was formed by slowly injecting the organic solution (1) into the aqueous solution (2) at 40 °C while continuously stirring. After complete homogenization, the water-miscible solvent (acetone and Tetra Hydro Furan) was evaporated using a rotary evaporator device at the National Research Centre, Cairo, Egypt, under reduced pressure at approximately 75 °C for 5 min.

#### 2.2.2. Preparation of Zn Phthalocyanine Encapsulated in the Nanoemulsion

To prepare the Zn phthalocyanine encapsulated NE, an organic solution (1) was initially prepared following the same method described in Section 2.2.1 [23,24]. Then, Zn phthalocyanine was dissolved in Miglyol 812 N oil at 40 °C under continuous stirring and added to the organic solution (1). In parallel, the homogeneous aqueous solution (2) was prepared following the same method used in the preparation of the nanoemulsion. The Zn phthalocyanine encapsulated in the nanoemulsion was obtained by slow injection of the solution containing Zn phthalocyanine and Miglyol 812 N oil that dissolved in solution (1) into the aqueous solution (2) at 40 °C with continuous stirring. The water-miscible solvent was then evaporated using a rotary evaporator device under reduced pressure at approximately 75 °C for 5 min.

#### 2.2.3. Physicochemical Characterization of NE) and NE + ZnPc

All formulations (NE and NE + ZnPc) underwent characterization based on their particle size distribution (mean size), polydispersity index (PDI) (a numerical value representing the homogeneity of the nanoemulsion), zeta potential and viscosity. The mean size and PDI of colloidal dispersions were determined at 22 °C using the Backscattered laser-amplified scattering reference method at an angle of 180°. The zeta potential was measured based on the electrophoretic mobility of nanoparticles between electrodes in an electric field [24]. All assays were carried out in triplicate.

#### 2.2.4. Morphological Characterization of Nanoemulsion (NE) and Zn Phthalocyanine Encapsulated in Nanoemulsion NE + ZnPc

The structure and morphology of the NE and NE + ZnPc were characterized by diluting concentrated nanoemulsion in distilled water (1:10) by placing a small drop of the diluted sample onto a holey film grid and allowing it to dry. The samples were then observed under a transmission electron microscope (TEM) to examine their structures and morphologies.

#### 2.2.5. Bacteria Strain and Culture Preparation

*S. aureus* was cultured on Tryptic Soy Agar-TSA, while *E. coli* was cultured on Luria-Bertani (LB) agar. Overnight cultures of *S. aureus* and *E. coli* were prepared by inoculating 10 mL of TSB and LB broth, respectively, followed by incubation at 37 °C. For the experiments, each culture was collected after serial dilution; *S. aureus* was diluted 1/10 (150 CFU/mL), while *E. coli* was diluted 1/100 (500 CFU/mL) due to its faster growth rate compared to *S. aureus* [25].

#### 2.2.6. The Effect of the Red Diode Light on *S. aureus* and *E. coli*

For each bacterial strain, 50 µL of *S. aureus* and *E. coli* were transferred separately to cell culture plates containing 6 wells each and then exposed to red diode light with an irradiance of 38.1 mW/cm^2^ [26] for varying time periods (1, 5, 10, 15 and 20 min). After exposure, 100 µL was taken from each well and diluted in 900 µL of distilled water, resulting in serial dilutions (ranging from 1/10 to 1/100,000). The diluted samples were then plated onto TSA and incubated aerobically at 37 °C for 24 h. The colony-forming units per milliliter (CFU/mL) were determined by counting the colonies on each plate.

#### 2.2.7. The Safety of Nanoemulsion on *S. aureus* and *E. coli* without Zn Phthalocyanine

A total of 50 µL aliquots of *S. aureus* and *E. coli* were transferred to a cell culture plate according to Table 1. The samples were incubated in the dark at room temperature for one hour and then mixed well using the vortex Heidolph reax top. After mixing, serial dilutions were made, and the resulting solutions were plated onto tryptic soy agar, as described previously. The colony counts on each plate were then quantified as the number of colony-forming units per milliliter (CFU/mL).

#### 2.2.8. Safety of Zn Phthalocyanine Encapsulated in Nanoemulsion (NE + ZnPc) Only on *S. aureus* and *E. coli*

As described in Section 2.2.7, the same protocol was followed with the exception that NE + ZnPc, which contained Zn phthalocyanine encapsulated in the nanoemulsion, was used instead of the NE only, as outlined in Table 2.

#### 2.2.9. Photodynamic Therapy against *S. aureus* and *E. coli*

Aliquots of 50 µL of *S. aureus* and *E. coli* were transferred to separate wells of a cell culture plate and then treated with different concentrations (500, 700, and 1000 µL) of the safety concentrations of Zn phthalocyanine encapsulated in the nanoemulsion and distilled water. After half an hour of incubation in the dark, the samples were exposed to red diode light for varying times, as indicated in Table 3 for *S. aureus* and Table 4 for *E. coli*. The samples were then mixed thoroughly, and serial dilutions were plated onto Tryptic Soy Agar. Colonies were counted as previously described.

#### 2.2.10. The Effect of the Incubation Time before the Photodynamic Therapy for *S. aureus* and *E. coli*

This experiment aimed to demonstrate the importance of incubation time between Zn phthalocyanine encapsulated in a nanoemulsion and each type of bacteria with distilled water prior to photodynamic therapy. For this, aliquots of 50 µL of *S. aureus* and *E. coli* were individually transferred to a cell culture plate and then added to the best concentration of NE + ZnPc of 1000 µL and distilled water. One group was directly exposed to red diode light without any incubation time (control), while the other groups were incubated for 30 min, 1 h, or 2 h with the prepared Zn phthalocyanine encapsulated in the nanoemulsion and each type of bacteria with distilled water before exposure to the red diode light, as shown in Table 5. After exposure, the samples were mixed well, and a serial dilution was made. Colonies of each plate were counted as previously mentioned.

## 3. Results

### 3.1. Preparation of Nanoemulsion and Zn Phthalocyanine Encapsulated in Nanoemulsion

**Figure 1:**
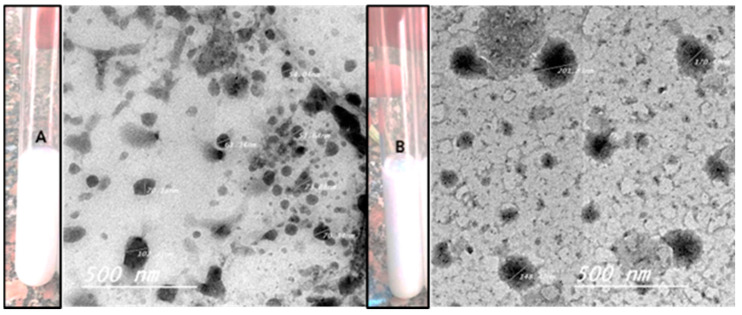
(**A**) TEM of NE and its morphology. (**B**) TEM of NE + ZnPc and its morphology.

The shape and color of the nanoemulsion produced and the nanoemulsion with phthalocyanine are shown in Figure 1.

### 3.2. The Morphological Characterization of Nanoemulsion (NE) and the Zn Phthalocyanine Encapsulated in Nanoemulsion NE + ZnPc

The morphology of NE and NE + ZnPc was characterized using transmission electron microscopy (TEM), as illustrated in Figure 1. The particle size of the nanoemulsion ranged from 30–500 nm (spherical in shape), while that of NE + ZnPc was within the range of 100–500 nm, consistent with the size distribution measurements presented in Figure 1 [27].

### 3.3. Physicochemical Characterization of Nanoemulsion (NE) and Zn Phthalocyanine Encapsulated in (NE + ZnPc)

**Figure 2:**
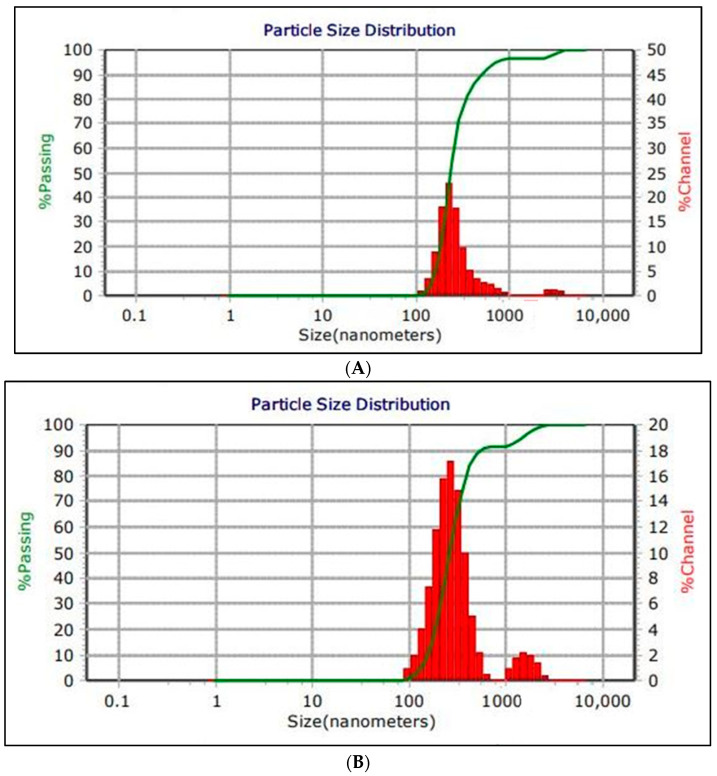
(**A**) Particle size of the nanoemulsion (NE) and (**B**) particle size of Zn phthalocyanine encapsulated in the nanoemulsion.

The results demonstrated that both NE and NE + ZnPc samples exhibited desirable nanometric size distributions, as shown in Figure 2. The PDI values for both samples were measured to be 0.0940. The average zeta potential for NE and NE + ZnPc was 51.47 mv and 47.32 mv, respectively, indicating a positive polarity for both. Additionally, the viscosity measurements for NE and NE + ZnPc were 0.952 and 0.9460, respectively, which is characteristic of low-viscosity NE.

### 3.4. The Effect of the Red Diode Light Only on S. aureus and E. coli

**Figure 3:**
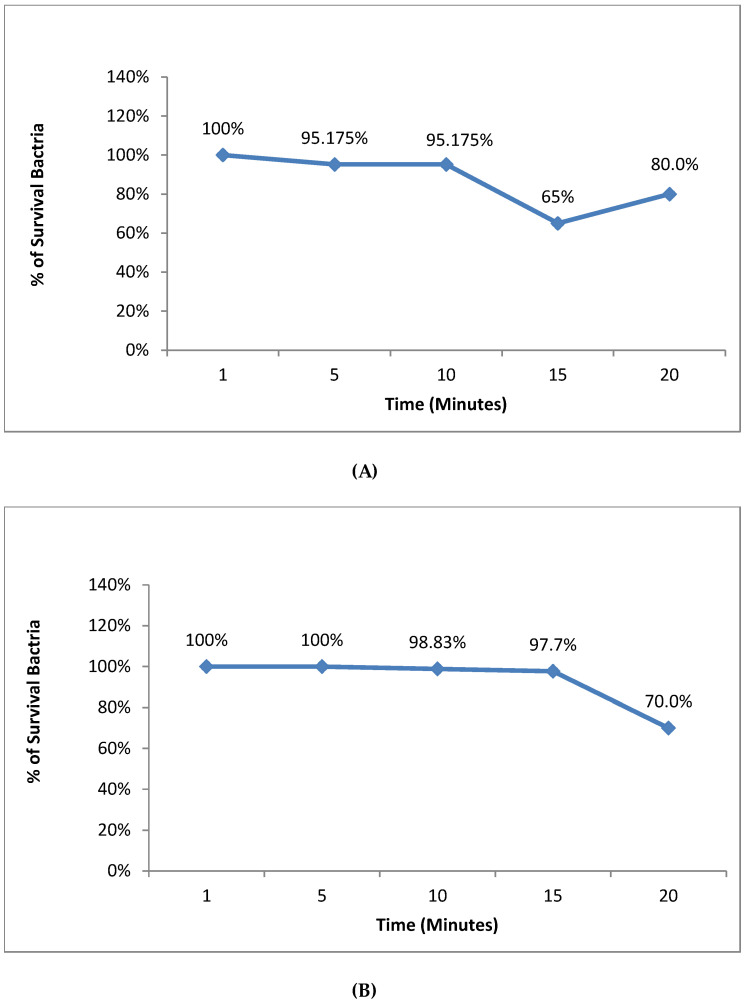
Effect of red light only on (**A**) *S. aureus* and (**B**) *E. coli*.

The results indicated that there was no significant light effect on the growth of *S. aureus* at 1 min. However, after 5 min, the bacterial survival percentage started to significantly decrease (*p* < 0.05), though, at 20 min, there was an increase in the bacterial survival percentage. In the case of *E. coli*, no significant light effect was observed on the bacterial survival percentage at 1 min or 5 min, but a significant decrease (*p* < 0.05) in the bacterial survival percentage was noted after 10 min, 15 min and 20 min (Figure 3).

### 3.5. Safety Evaluation of NE and NE + ZnPc on S. aureus and E. coli

**Figure 4:**
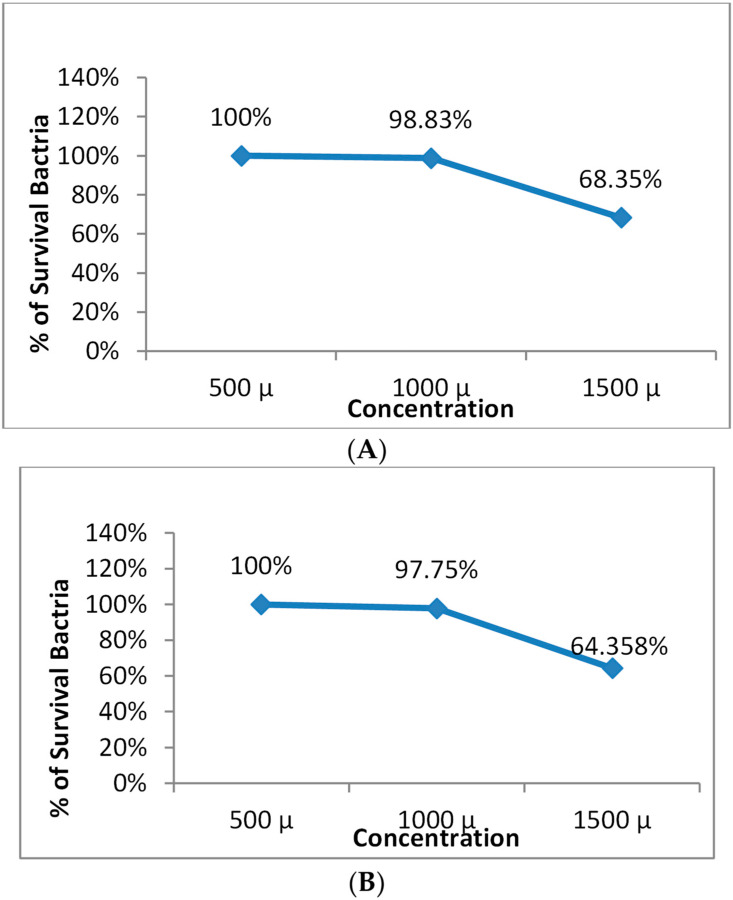
Safety of the nanoemulsion on (**A**) *S. aureus* and (**B**) *E. coli*.

**Figure 5:**
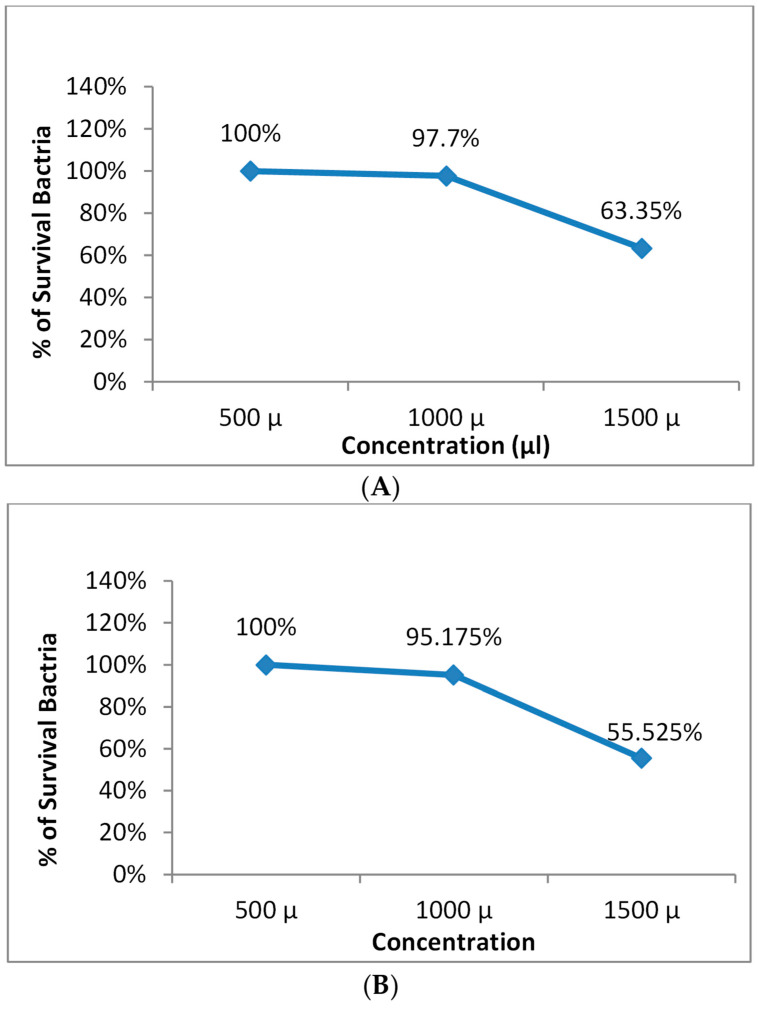
Safety of the nanoemulsion + Zn Phthalocyanine on (**A**) *S. aureus* and (**B**) *E. coli*.

Figure 4 and Figure 5 demonstrate that the prepared nanoemulsion and the prepared nanoemulsion + Zn phthalocyanine did not exhibit toxicity to cells at concentrations of 500 µL, 700 µL or 1000 µL.

### 3.6. Photodynamic Therapy against S. aureus and E. coli

**Figure 6:**
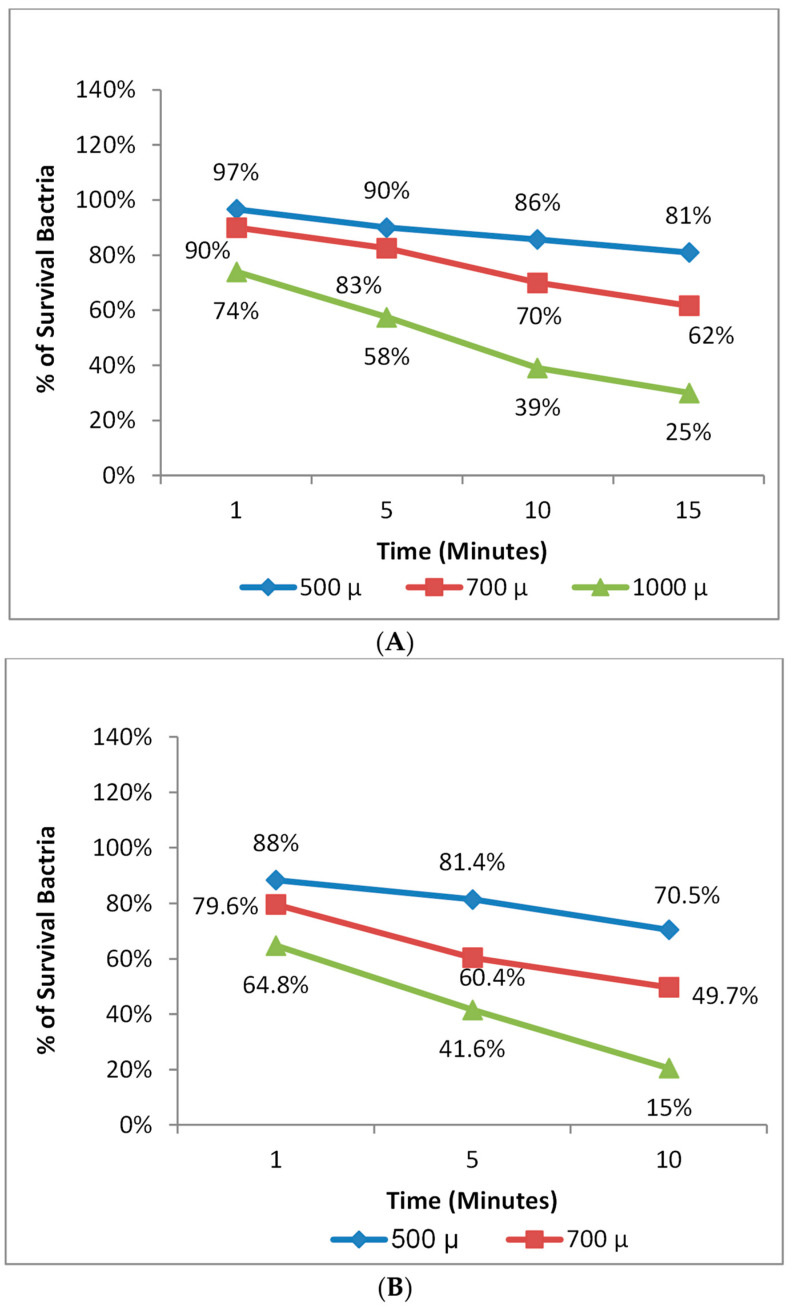
Photodynamic therapy on (**A**) *E. coli* and (**B**) *S. aureus*.

The highest antimicrobial efficiency was achieved by applying antimicrobial photodynamic therapy using nanoemulsion + Zn phthalocyanine at a concentration of 1000 µL for 10 min and 15 min, with 85% and 75% effectiveness against *S. aureus* and *E. coli*, respectively (Figure 6).

### 3.7. The Effect of Incubation Time before Photodynamic Therapy for S. aureus and E. coli

**Figure 7:**
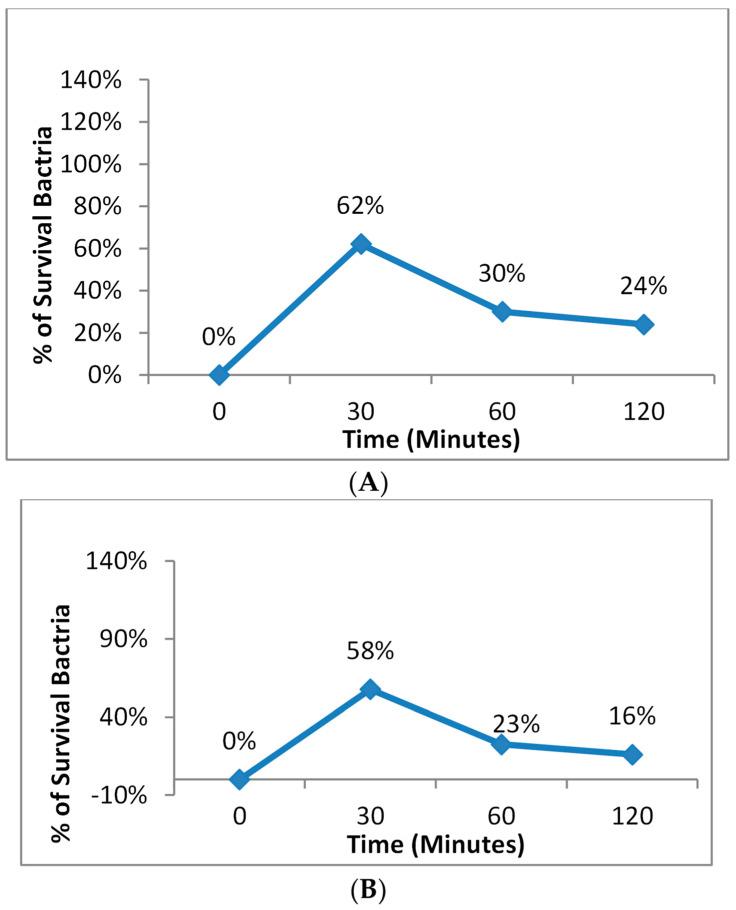
The Effect of an Increase in the Photodynamic therapy on (**A**) *E. coli* and (**B**) *S. aureus*.

The incubation time between the Zn phthalocyanine encapsulated in nanoemulsion with *E. coli* and *S. aureus* before antimicrobial photodynamic therapy was found to be crucial. Without this incubation time, there was no inhibition in cell number. Increasing the incubation time led to a higher bactericidal effect, as shown in Figure 7.

## 4. Discussion

Multi-drug resistant bacteria developed resistance to penicillin antibiotics through the expression of the blaZ gene encoding β-lactamase, which is synthesized when staphylococci are exposed to β-lactam antibiotics [28,29]. Resistance to methicillin is attributed to the mecA gene responsible for methicillin resistance [30], while fluoroquinolone and quinolone resistance emerged through the stepwise acquisition of chromosomal mutations for the treatment of gram-negative bacterial infections, followed by the gram-positive bacterial spectrum [31]. Resistance to vancomycin appeared in VRSA strains due to the acquisition of vanA, which alters peptidoglycan biosynthesis. The emergence of antibiotic-resistant bacteria has become a major global health problem. Photodynamic therapy has been proposed as an alternative to conventional treatments for eradicating multidrug-resistant bacteria without inducing resistance, as the ROS generated during the photodynamic reaction can damage multiple cellular structures, from the membrane to organelles, reducing the likelihood of developing PDT-resistant strains [32].

The study approach was to encapsulate a hydrophobic photosensitizer drug (ZnPc) in an oil-in-water (O/W) nanoemulsion as a drug carrier using Miglyol 812N oil (triglyceride ester of saturated coconut) and use this prepared nanoemulsion with Zn phthalocyanine as an antimicrobial photodynamic therapeutic agent to control multidrug-resistant bacteria. The efficiency of antimicrobial photodynamic therapy can be greatly enhanced based on the photosensitizer type and the delivery system’s presence.

Phthalocyanine was chosen as the preferred photosensitizer, a second-generation photosensitizer, due to its higher oxidative power and its ability to produce singlet oxygen effectively [33,34]. Phthalocyanines are commercially available and are non-toxic in the dark, with rapid elimination and minimal side effects at low concentrations [35]. Their chemical structure allows them to associate with several metals, such as Zn, Cu, and Mg, which, in turn, enhances their ionic characteristics and phototoxicity [36].

In this study, Zn phthalocyanines were chosen as the photosensitizer for aPDT applications due to their efficient encapsulation in nanoemulsions, making them better than other photosensitizers such as Aluminum chloride phthalocyanine entrapped in nanoemulsion, as reported in previous studies [37,38,39]. However, the hydrophobic nature of ZnPc increases the likelihood of aggregation, making it crucial to use an organic solvent or delivery system to maintain its hydrophobicity and enhance its efficiency in antimicrobial photodynamic therapy [26].

Previous studies utilized photosensitizers dissolved in aqueous media such as saline or DMSO, but these media were reported to be unstable and could cause damage to tissues and an increase in host cell permeability [40]. Hence, the use of a stable delivery system such as nanoemulsion was preferred as it offers high stability, bioavailability, and penetration, as well as preventing the accumulation of hydrophobic photosensitizer to enhance antimicrobial photodynamic therapy [41].

The particle size of the nanoemulsion is critical for intravascular delivery, as the diameter of the smallest blood capillaries in the human body is in the 4–7 µm range, and larger particles may cause capillary occlusion. TEM measurements were performed to determine the size, morphology and size distribution of the prepared nanoemulsion. The results showed a nanometric size range of 30–500 nm for both the NE and NE + ZnPc formulations, as shown in Figure 3. The polydispersity index (PDI) was used to assess sample quality, with a PDI below 0.4 indicating homogeneity in particle size and a PDI over 1 indicating complete heterogeneity [42]. The results showed a PDI of 0.0940, indicating excellent sample quality with a narrow size distribution, making it a perfect delivery system for dissolving hydrophobic photosensitizers.

The Zeta potential is a crucial factor for predicting the dispersion stability of nanoemulsions and measuring the surface charge of particles when suspended in a liquid. The value of Zeta potential depends on the physicochemical properties of the system. A positive zeta potential value of ±30 mV or higher is generally considered sufficient for ensuring the physical stability and long lifetime of the nanoemulsion [43]. In our study, the Zeta potential values of the nanoemulsion and the nanoemulsion with phthalocyanine were 51.47 mV and 47.32 mV, respectively. Additionally, a good affinity of ZnPc for the magnetic nanoemulsion with an average value of 54.47–150 mV was observed [44].

Viscosity is another crucial parameter for characterizing the physicochemical properties of nanoemulsions. It helps determine whether the system is an oil-in-water (O/W) or water-in-oil (W/O) nanoemulsion. Low viscosity indicates that it is an O/W type, while high viscosity indicates a W/O type nanoemulsion. Viscosity measurements for our prepared nanoemulsions were less than 21 cP or 30 cP, with a minimum viscosity of 10.68 cP reported in previous studies [45,46,47]. In our study, the viscosity of the nanoemulsion and the nanoemulsion with ZnPc were 0.952 and 0.9460, respectively, which suggests that the prepared nanoemulsion was a perfect O/W system. This is important for the efficient encapsulation of hydrophobic photosensitizers in antimicrobial photodynamic therapy applications, as reported in other studies [48].

To ensure that the nanoemulsion and phthalocyanine encapsulated in the nanoemulsion, as well as red diode light, had no bactericidal effects on bacteria, repeatable dark toxicity experiments were conducted. Specific concentrations of the non-toxic effects of the nanoemulsion and Zn phthalocyanine encapsulated in the nanoemulsion alone were determined to be 500, 700 and 1000 µL, and the minimal emission time with red diode light alone that had no bactericidal effect was determined. The results showed that toxic products (ROS) of antimicrobial photodynamic therapy (aPDT) were only generated when the photosensitizer was excited by a light source of a specific wavelength of 625 nm. This restricted oxidative damage to the site of the photosensitizer [32].

Our dark toxicity experiments showed that the minimal safety time for red diode light alone that kept bacteria growth in *S. aureus* was 10 min, while in *E. coli*, it was 15 min. In previous studies, it was reported that the minimal safety time for red diode light alone that kept bacteria growth in *S. aureus* was also 15 min, whereas in *E. coli*, it was 30 min [49]. This suggests that *E. coli* required more time to be killed due to the complexity of its cell wall structure, which is more complex than that of *S. aureus*, as shown in Figure 2 [12]. However, in our study, the bacterial growth increased in *S. aureus* after 20 min. This may be caused by a mutation, which may have increased the bacterial resistance [49]. The bactericidal effect only appeared by uptaking Zn phthalocyanine encapsulated in the nanoemulsion (1000 µL) in *S. aureus* and *E. coli* with red diode light produced reactive oxygen species that caused oxidative damage sufficient to kill multidrug-resistant bacteria.

The antimicrobial photodynamic therapy resulted in a photokilling percentage of 85% and 75% for *S. aureus* and *E. coli*, respectively, as shown in Figure 6. This effect was more pronounced in *S. aureus* compared to *E. coli*. The bactericidal effect against *S. aureus* was more effective than against *E. coli* due to differences in their cell membrane structures, as discussed previously and demonstrated in Figure 2 [17]. Other studies have also used antimicrobial photodynamic therapy with different methods and reported varying results. One study induced a >4–5 log decreases in the microbial population without nanoemulsion [50], while another used two new zinc phthalocyanine derivatives, ZnPc1 and ZnPc2, to observe photoinactivation responses in *E. coli* and *S. aureus* [51]. Additionally, other studies reported photokilling activities in *S. aureus*, *E. coli* and *Candida albicans* [52].

The incubation time of leaving the prepared Zn phthalocyanine encapsulated in nanoemulsion with bacteria in the dark before the antimicrobial photodynamic therapy is critical, and about half an hour is required as the optimum incubation period. Without this incubation time, there was no inhibition in the cell number. This incubation time allowed Zn phthalocyanines encapsulated in the nanoemulsion to easily enter strange cells such as multidrug-resistant bacteria, which then underwent antimicrobial photodynamic effects after exposure to red diode light. However, in case of longer incubation than 30 min, the cells’ mortality is caused by the toxicity of Zn phthalocyanines encapsulated in the nanoemulsion and not due to applying photodynamic therapy. The uptake of phthalocyanine (Pc) was observed on the cell wall as well as in the cytoplasm of the HA-MRSA after three hours of incubation, as reported in [53]. Another study demonstrated cellular uptake of photosensitizer by bacteria after one hour [54].

This study showed an interesting alternative method that maintains the hydrophobicity of the photosensitizer and allows it to function effectively by encapsulating it in the nanoemulsion in the nanometric range. This presents a synergistic approach for future trials in aPDT. Photodynamic therapy (PDT) has been recently demonstrated to be a powerful strategy for tumor destruction, and nanotechnology is also suggested to reduce treatment dosage and frequency by enabling the delivery of drugs within confined tissues using nanoemulsions [55]. Several innovative alternatives, such as ultrasonic irrigation, laser wavelengths, and photodynamic therapy, have been used for the treatment of oral infections caused by microorganisms that reside within the root canal system [47]. The COVID-19 pandemic, caused by a new coronavirus, SARS-CoV-2, has spread rapidly across the world [56]. Its severity necessitates the development of new methods for the prevention and treatment of the disease [57]. Antiviral photodynamic therapy is one approach that has been shown to completely block viral replication within infected cells during the phase of active viral synthesis, as reported in [58]. Photodynamic therapy has also been used in the treatment of bacterial skin infections [54].

## 5. Conclusions

This study introduces a novel approach for using hydrophobic photosensitizers in aqueous media. The process involves encapsulating Zn phthalocyanine in a nanoemulsion using solvents, essential oils, biopolymers, lipophilic surfactants and hydrophilic surfactants. The resulting nanoemulsion is well-characterized, with a size range of 30–500 nm, and is stable at room temperature for an extended period. This approach has been shown to be effective in antimicrobial photodynamic therapy, with a photokilling percentage of 85% and 75% against *S. aureus* and *E. coli*, respectively. This successful formation of Zn phthalocyanine encapsulated in nanoemulsion opens the door for the use of hydrophobic photosensitizers and drugs in various biological and medical applications. Future research will focus on investigating the positive effects of Zn phthalocyanine encapsulated in nanoemulsion as an antimicrobial photodynamic therapy agent for oral, skin, dental and cancer treatment.

## Figures and Tables

**Table 1:** The different concentrations of nanoemulsion in mixture with *S. aureus* or *E. coli* without Zn phthalocyanine.

Treatments	Control	1	2	3
*S. aureus* or *E. coli* (µL)	50	50	50	50
Concentration of NE (µL)	0	500	1000	1500
Distilled water (µL)	1950	1450	950	450

**Table 2:** The different concentrations of Zn phthalocyanine encapsulated in nanoemulsion (NE + ZnPc) only on *S. aureus* or *E. coli*.

Treatments	Control	1	2	3
*S. aureus* or *E. coli* (µL)	50	50	50	50
Concentration of NE + ZnPc (µL)	0	500	1000	1500
Distilled water (µL)	1950	1450	950	450

**Table 3:** Photodynamic therapy against *S. aureus*.

Photodynamic Therapy Duration (min)	0 (Control)	1	5	10
Concentration of *S. aureus* (µL)	50	50	50	50
Concentration of NE + ZnPc (µL)	0	500	700	1000
Distilled water (µL)	1950	1450	1250	950

**Table 4:** Photodynamic therapy against *E. coli*.

Photodynamic Therapy Duration (min)	0 (Control)	1	5	10	15
The concentration of *E. coli* (µL)	50	50	50	50	50
Concentration of NE + ZnPc (µL)	0	500	700	1000	1000
Distilled water (µL)	1950	1450	1250	950	950

**Table 5:** The effect of the incubation time before the photodynamic therapy for *S. aureus* and *E. coli*.

Incubation Time (min)	0 (Control)	30 min	60	120
*S. aureus* or *E. coli* (µL)	50	50	50	50
Concentration of NE + ZnPc (µL)	0	1000	1000	1000
Distilled water (µL)	1950	950	950	950

## Data Availability

The data presented in this study are available on request from the corresponding author. The data are not publicly available due to privacy.
